# Characterization of the *Taenia *spp HDP2 sequence and development of a novel PCR-based assay for discrimination of *Taenia saginata *from *Taenia asiatica*

**DOI:** 10.1186/1756-3305-3-51

**Published:** 2010-06-11

**Authors:** Luis M González, Begoña Bailo, Elizabeth Ferrer, Maria D Fernandez García, Leslie JS Harrison, Michael RE Parkhouse, Donald P McManus, Teresa Gárate

**Affiliations:** 1 Parasitology Department, Centro Nacional de Microbiología, Instituto de Salud Carlos III, 28220 Majadahonda, Madrid, Spain; 2 BIOMED and Parasitology Department, Universidad de Carabobo Sede Aragua, Maracay, Venezuela; 3 Sir Alexander Robertson Building, The University of Edinburgh, Easter Bush Veterinary Centre, Easter Bush, Roslin, Midlothian, Scotland, EH25 9RG UK; 4 Instituto Gulbenkian de Ciencia, R. Quinta Grande 6, Oerias, Portugal; 5 Molecular Parasitology Laboratory, Australian Centre for International and Tropical Health, Queensland Institute of Medical Research, The Bancroft Centre, 300 Herston Road, Brisbane, Queensland 4029, Australia

## Abstract

A previously described *Taenia saginata *HDP2 DNA sequence, a 4-kb polymorphic fragment, was previously used as the basis for developing PCR diagnostic protocols for the species-specific discrimination of *T. saginata *from *T. solium *and for the differentiation of *T. saginata *from *T. asiatica*. The latter was shown subsequently to lack the required specificity, so we undertook genetic studies of the HDP2 sequence from *T. saginata *and *T. asiatica *to determine why, and to develop a novel HDP2-PCR protocol for the simultaneous unambiguous identification of human taeniids. Sequencing and further analysis of the HDP2 DNA fragments of 19 Asiatic isolates of *T. saginata *and *T. asiatica *indicated that the HDP2 sequences of both species exhibited clear genomic variability, due to polymorphic variable fragments, that could correspond to the non-transcribed region of ribosomal DNA. This newly observed polymorphism allowed us to develop a novel, reproducible and reliable HDP2-PCR protocol which permitted the simultaneous discrimination of all *T. saginata *and *T. asiatica *isolates examined. This species-specific identification was based on, and facilitated by, the clear size difference in amplicon profiles generated: fragments of 1300 bp, 600 bp and 300 bp were produced for *T. asiatica*, amplicons of 1300 bp and 300 bp being obtained for *T. saginata*. Control *T. solium *samples produced one amplicon of 600 bp with the HDP2-PCR protocol. The assay has the potential to prove useful as a diagnostic tool in areas such as South East Asia where *T. saginata*, *T. asiatica *and *T. solium *coexist.

## Findings

Human taeniasis results from intestinal infection with the adult tapeworms *Taenia saginata*, *Taenia asiatica *or *Taenia solium*. Ingestion of eggs of *T. saginata *and *T. solium/ T. asiatica *causes cysticercosis in cattle and pigs, respectively. Importantly, *T. solium *eggs can also infect man, giving rise to human cysticercosis, with the frequent localization of the metacestodes in the brain, causing potentially fatal neurocysticercosis (NCC) [1-3]. Infection with *T. solium *is therefore a serious public health problem, notably in endemic areas (Latin America, Africa, Asia), but also in non-endemic areas due to imported cases [4]. Hence, and as an essential part of transmission control programs, there is an immediate and practical need for reliable and species-specific diagnosis of human taeniasis caused by *T. saginata*, *T. asiatica *and *T. solium *in order to identify tapeworm carriers, particularly those with *T. solium*, thus helping to avoid transmission of cysticercosis/NCC.

Until recently, species-specific diagnosis of taeniasis was unsatisfactory. Conventional clinical and morphological identification has low specificity and sensitivity [5]. Moreover, adult worms of *T. saginata *and *T. asiatica *are frequently confused due to their morphological similarities [6]. Copro-antigen diagnosis by ELISA, that detects parasite antigens in patient feces, is based on the use of polyclonal antibody reagents although it does not distinguish among the human intestinal taeniids [7,8]. More recently, serological diagnosis for *T. solium *taeniasis has been reported with recombinant ES antigens [9,10], but this assay not necessarily indicates active infection.

Various PCR approaches have been developed for species-specific identification of DNA from *T. saginata, T. asiatica *and *T. solium *[6,11-26]. Thus, the previously described *T. saginata *HDP2 DNA sequence [12,27], a 4-kb polymorphic fragment, was the basis for various PCR-based diagnostic protocols for the species-specific detection of *T. saginata *and *T. solium *taeniasis [12,17,25,26], as well as for the specific differentiation of *T. saginata *and *T. asiatica *samples [22]; however, the developed HDP2-PCR protocol was later shown to lack the required specificity to discriminate *T. saginata *from *T. asiatica*.

Although the genomic structure of the HDP2 fragment had been partially described for *T. saginata *and *T. solium *[12], its homologue in the taxonomically closely related *T. asiatica *had not [19,28-32]. Therefore, the aims of this study were: (i) to undertake genetic analysis of the HDP2 DNA sequence [12,27] from *T. saginata *and *T. asiatica *isolates, and (ii) to develop a novel HDP2-PCR protocol for the simultaneous species-specific identification of *T. saginata *and *T. asiatica*, using a total of 19 taeniid cestode proglottid isolates from taeniasis patients of Asian origin (Table 1). Morphological identification of the proglottid samples was based on the number of uterine branches, and confirmed by genetic characterization, according to published protocols [5,19]. Genomic DNA (gDNA) was extracted from the ethanol-preserved proglottids samples using the method described by Sambrook et al. [33]. Two purified gDNA samples from *T. solium *(Venezuelan and Mexican isolates) and one from *T. saginata *(Spanish isolate) were used as positive controls.

**Table 1:** Geographical origin of the 6 *Taenia saginata *and 13 *Taenia asiatica *samples analyzed^a^.

Sample origin	Sample code number and species*	
	
	*Taenia saginata*	*Taenia asiatica*
Indonesia		**#12**
Korea		**#16**, #**19**
Philippines		#7, #11
Taiwan	#4, #8, #10, #14	#1, **#3**, #6, #9, #13, #15, **#17**, #18
Thailand	#2	
Asia (country unknown)	#5	

^a^All samples were identified to species level by two separate PCRs; a multiplex PCR [6] and the novel HDP2-PCR III described in this study. The numbers shown in bold correspond to samples that could not be differentiated by the HDP2-multiplex-PCR [22].

**Figure 1:**
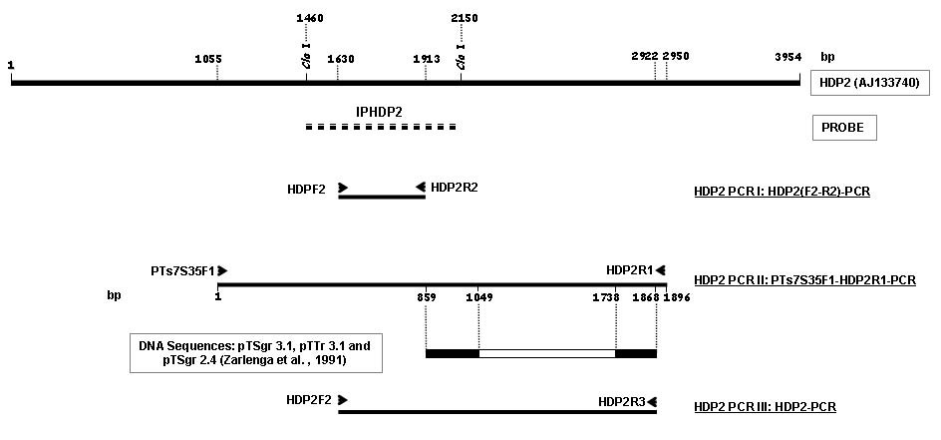
**Strategy for amplification and verification of the HDP2 sequences**. The location of probe IHDP2 used in Southern-blots is indicated by a discontinuous line below the *T.saginata *HDP2 sequence [GenBank: AJ133740]. The locations of the oligonucleotide primers within the *T. saginata *HDP2 DNA sequence [GenBank AJ133740] for the different molecular protocols used, and the directions of these are indicated by arrows: HDP2F2 and HDP2R2 (PCR I: HDP2 (F2-R2)-PCR); PTs7S35F1 and HDP2R1 (PCR II: PTs7S35F1-HDP2R1-PCR); HDP2F2 and HDP2R3 (PCR III: HDP2-PCR). The location of the sequences with similarity to the *T. saginata *and *T. asiatica *partial HPD2 DNA fragments (PTs7S35F1-HDP2R1 amplicon), pTSgr 3.1, pTSgr 2.4 and pTTr 3.1, are indicated by the black area of the bar. The restriction enzyme sites are indicated by the lines.

Previously described multiplex PCRs were used to amplify the gDNA obtained from the 19 Asian taeniid proglottids [6,22] and the positive controls. In addition, the gDNAs from the 19 Asian samples were examined by three new HDP2-based PCRs, using HDP2-derived primers (Fig. 1, table 2). HDP2 PCRs I and II were used to analyze the genomic characteristics of the HDP2 DNA sequence within the Asiatic taeniid isolates, while the novel HDP2 PCR III was developed to differentiate the Asian samples. Table 2 shows the PCR conditions and the sequence of primers used.

**Table 2:** PCR names, primer sequences and amplification conditions used.

PCR primer name	Primer sequence	Amplification conditions (Taq polymerase; BIOTOOLS , Spain)
HDP2 PCR I: HDP2F2 HDP2R2	5'-GCTGTACCAGCACCTAACCRTCC-3' 5'-GCACACCGCAGCCAATTGGCTG-3'	94°C for 30 sec, 55°C for 30 sec, 72°C for 1 min, 72°C for 7 min (35 cycles)
HDP2 PCR II: PTs7S35F1 HDP2R1	5'-CAGTGGCATAGCAG AGGAGGAA-3' 5'-GCGGAAAGTGGATCCGACTTCGATGG-3'	94°C for 30 sec, 56.5°C for 30 sec, 72°C for 2 min, 72°C for 7 min (35 cycles)
HDP2 PCR III: HDP2F2 HDP2R3	5'-GCTGTACCAGCACCTAACCRTCC-3' 5'-ATCCTGCTCCATGTCGCTTCTAGC-3'	94°C for 30 sec, 60°C for 30 sec, 72°C for 2 min, 72°C for 7 min (35 cycles)

To study the HDP2 DNA sequence organization in the 19 Asian *T. saginata *and *T. asiatica *isolates, 5 μg of each parasite gDNA was digested to completion with *Cla *I (Roche, Penzberg, Germany), as recommended by the manufacturer. Electrophoresis, Southern blotting, probe labeling, and hybridization procedures were carried out, with minor modifications, as previously described [12]. The probe used was one fragment derived from the HDP2 sequence, previously named IPHDP2 [12], which is a variable fragment from the mid region of HDP2 (Fig. 1).

In addition, the HDP2 DNA sequence of the *Taenia *isolates were PCR-amplified with the PTs7S35F1/ HDP2R1 primers set (Fig. 1, Table 2), using 10 ng of gDNA from each parasite sample. After electrophoresis, the amplicons were visualized and purified using a QIAquick Gel Extraction Kit (Qiagen,, Valencia, CA). All the DNA fragments were automatically sequenced by standard Sanger chemistry using a Model 377 ABI PRISM system. We used a primer walking sequencing strategy involving PTs7S35F1, HDP2F2, HDP2R2 forward primers and HDP2R2, HDP2R1 reverse primers (Fig. 1). The DNA sequences obtained and their alignments were analyzed by Seqman II (DNASTAR) and Clustal W2 [34] software packages. DNA sequence comparisons were carried out using the GenBank databases and BLASTn [35]. The nucleotide sequences obtained from amplification of the HDP2 gDNA fragments of the *Taenia *isolates have been deposited in the EMBL/GenBank databases under the following accession numbers: FM212953-71.

**Figure 2:**
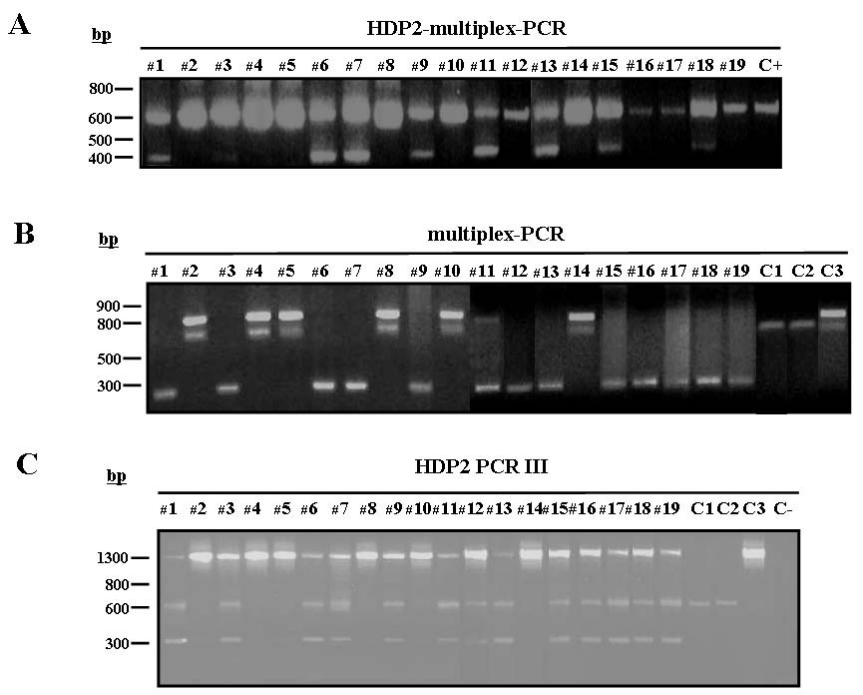
**(A) Differential diagnosis of *T. saginata *and *T. asiatica *isolates from Asia using the HDP2-multiplex-PCR **[22]. Lanes 1, 3, 6, 7, 9, 11, 12, 13, 15, 16, 17, 18, 19, *T. asiatica*; lanes 2, 4, 5, 8, 10, 14, *T. saginata*.; C+, *T. saginata *control sample (Spanish origin). Amplification products were fractionated by electrophoresis in a 1% (w/v) agarose gel and stained with ethidium bromide. The numbers on the left indicate the sizes (in bases pairs, bp) of molecular weight markers. **(B) **Differential diagnosis of *T. saginata *and *T. asiatica *isolates from Asia by multiplex-PCR [6]. Lanes 1, 3, 6, 7, 9, 11, 12, 13, 15, 16, 17, 18, 19, *T. asiatica*; lanes 2, 4, 5, 8, 10, 14, *T. saginata*. C1 control sample (*T. solium *Venezuelan origin); C2 control sample (*T. solium *Mexican origin); C3 control sample (*T. saginata *Spanish origin). Amplification products were fractionated by electrophoresis in a 2% (w/v) agarose gel and stained with ethidium bromide. The numbers on the left indicate the sizes (in bases pairs, bp) of molecular weight markers**. (C) **Differential diagnosis of *Taenia *spp. DNA samples using the HDP2 PCR III. 10 ng gDNA from Asian isolates of *T. asiatica *(lanes 1, 3, 6, 7, 9, 11, 12, 13, 15, 16, 17, 18 and 19); Asian isolates of *T. saginata *(lanes 2, 4, 5, 8, 10 and 14); *T. solium *(Venezuelan and Mexican origin, C1 and C2); and *T. saginata *(Spanish origin, C3) were amplified by HDP2-PCR. The amplification products were fractionated by electrophoresis in a 1% (w/v) agarose gel and stained using ethidium bromide. The numbers on the left indicate the sizes (in bases pairs, bp) of molecular weight markers.

Our analysis showed that the Asian samples could clearly be differentiated using the multiplex PCR described by Yamasaki et al. [6], but not by the HDP2-based multiplex PCR (Fig. 2a/2b) reported by Gonzalez et al. [22], as samples #3, #12,#16,#17 and #19 were incorrectly identified as *T. saginata*. This finding prompted us to undertake further analysis of the genomic structure and sequence analysis of the HDP2 DNA fragments from the Asian taeniid isolates as now described.

**Figure 3:**
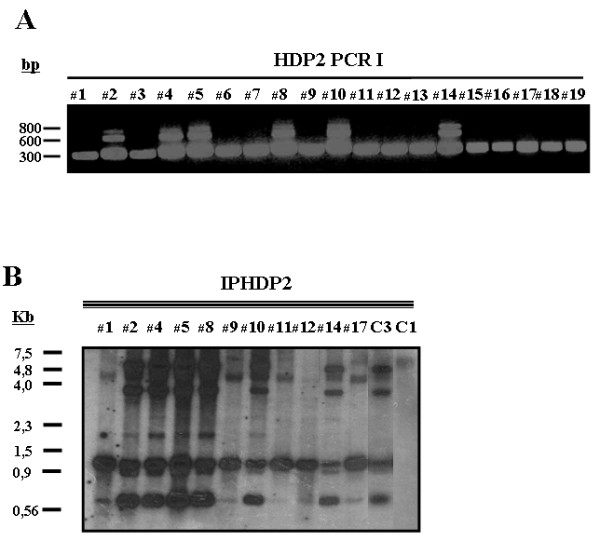
**Polymorphism in the HDP2 sequences**. **(A) **HDP2 PCR I using 10 ng gDNA from Asian isolates of *T. asiatica *(lanes 1, 3, 6, 7, 9, 11, 12, 13, 15, 16, 17, 18 and 19); and Asian isolates of *T. saginata *(lanes 2, 4, 5, 8, 10 and 14). **(B) **Southern-blot analysis. 5 μg gDNA from Asian isolates of *T. asiatica *(lanes 1, 9, 11, 12 and 17); Asian isolates of *T. saginata *(lanes 2, 4, 5, 8, 10 and 14); *T. solium *(Venezuelan origin, C1); and *T. saginata *(Spanish origin, C3) were digested to completion with *Cla *I and probed with the digoxigenin-labeled *T. saginata *IPHDP2 probe.

After *Cla *I digestion of gDNAs from the Asian isolates, *T. saginata *and *T. solium *controls, and IPHDP2 probe hybridization as described above, different restriction enzyme product profiles were identified (Fig. 3b). With *T. saginata*, bands of 4.5, 3, 2, 1 and 0.7 kb were produced, whereas with *T. asiatica *bands of 4, 1 and 0.7 kb were obtained. As an alternative and complementary approach for studying the molecular organization of the HDP2 sequence, *T. saginata *and *T. asiatica *gDNA samples were amplified with primers derived from the IHDP2 probe (HDP2 PCR I); three amplicons for *T. saginata *were observed, but only one for *T. asiatica *(Fig. 3a). Taking these results together, *T. saginata *and *T. asiatica *showed polymorphism in their HDP2 DNA sequences.

To explore the contribution of nucleotide variability to the observed HDP2 sequence polymorphism, a fragment of 1896 bp from the IHDP2 region was obtained from all 19 Asian taeniid DNA samples using the HDP2 PCR II protocol. A comparison of all the IHDP2 fragments obtained [GenBank: FM212953-FM212971] by Clustal W2, revealed a high degree of homology, particularly in regard to the *T. saginata *isolates. Thus, alignment of the 1896 bp fragment from the control *T. saginata *HDP2 sequence [GenBank: AJ133740] with the amplified fragments from the Asian *T. saginata *samples yielded nucleotide identities from 99.6- 99.7%. Alignment of the control *T. saginata *HDP2 sequence with the *T. asiatica *fragments yielded 97.0-98.8% nucleotide identity. This, the Asian *T. asiatica *HDP2 fragments showed a sequence divergence of 0.1% to 2.1%, compared with divergence of 0.0-0.1% recorded for the Asian *T. saginata *isolates and the control Spanish *T. saginata *isolate, thereby confirming low nucleotide variability between the two cestode taxa. Consequently, this structural analysis suggested that the HDP2 sequence polymorphism could be explained by variable representation of partially repeated sequences in the mid region of the HDP2 fragment, IHDP2, rather than due to nucleotide sequence divergence.

Seqman II-alignment of the partial HDP2 DNA fragments for *T. saginata *and *T. asiatica *within the NTR of the rDNA sequences of *T*. *saginata *(pTSgr 3.1, pTSgr 2.4) and *T. asiatica *(pTTr 3.1) [21,36], revealed that the sequences were identical. Accordingly, the HDP2 sequence could thus also represent a variable repetitive fragment, being part of the NTR from rDNA. Notably, the identities among the sequences were restricted to nucleotides 859-1049 and 1738-1868 of the HPD2 DNA plus strand; pTSgr 3.1 and pTTr 3.1 has a 0.7 kb internal fragment, whereas pTSgr 2.4 does not (Fig. 1). This genetic variability in the NTR of the rDNA of *T. saginata *and *T. asiatica *allowed us to design a PCR protocol (HDP2 PCR III) for the simultaneous differential identification of the two species (Fig. 1). The HDP2F2/HDP2R3 primer set was designed from this region in the mid variable central part of HDP2 (Fig. 1).

Using 10 ng of gDNA from the Asian isolates and control samples, and following the PCR conditions described in Table 2, unambiguous species-specific amplification was obtained for all the samples analyzed (Fig. 2c). Three amplicons were evident with the *T. asiatica *samples (1300 bp, 600 bp and 300 bp), and two amplicons were generated for *T. saginata *(a strong band of 1300 bp and a weak band of 300 bp); the control *T.solium *samples produced one amplicon (600 bp) (Fig. 2c). Therefore, the novel HDP2 PCR III yielded results similar to those of Yamasaki et al. [6] (Fig. 2b). These assays will prove particularly useful in areas such as South East Asia where *T. saginata*, *T. asiatica *and *T. solium *coexist [37,38].

In conclusion, this study showed and defined genetic polymorphism in the HDP2 sequence of the human taeniid cestodes *T. saginata *and *T*. *asiatica*. An unexpected but, nevertheless notable finding, was that HDP2 may be a repetitive fragment of the NTR within taeniid rDNA. This could explain the success of the published PCR protocols derived from this sequence in the sensitive diagnosis of taeniasis and NCC [12,13,17,25,26,39]. These findings were used to design a new HDP2 based PCR protocol, which demonstrated to be useful for the unambiguous discrimination of *T. saginata *from *T. asiatica*.

## Abbreviations

NCC: neurocysticercosis; ELISA: enzyme-linked immunosorbent assay; PCR: Polymerase chain reaction; ES: excretion-secretion; DNA: Deoxyribonucleic acid; DNAg: genomic DNA; rDNA: ribosomal DNA; NTR: non-translated region; HDP2: a 4- kb polymorphic DNA fragment.

## Competing interests

The authors declare that they have no competing interests.

## Authors´contributions

LMG, LJSH, RMEP, DPM and TG conceived and designed the experiments.: LMG, BB, EF and MDFG performed the experiments. LMG, EF, DPM and TG analyzed the data. LMG, TG, LJSH, MREP and DPM wrote the paper. All authors read and approved the final manuscript.
